# Dr. Bowling of the Nashville Journal

**Published:** 1858-01

**Authors:** 


### Dr. Bowling of the Nashville Journal.

The inconsistency, unfairness, and double-dealing of the individual whose name heads this article, having been established, *beyond controversy*, we shall not pursue him into the mire and filth under which he has *taken cover*, but should he again emerge from his *retreat*, and *stray* into the confines of decency, we may be tempted to re-pepper his vulgar carcass.

